# Involvement of the MGF 110-11L Gene in the African Swine Fever Replication and Virulence

**DOI:** 10.3390/vaccines11040846

**Published:** 2023-04-14

**Authors:** Vivien Tamás, Cecilia Righi, István Mészáros, Federica D’Errico, Ferenc Olasz, Cristina Casciari, Zoltán Zádori, Tibor Magyar, Stefano Petrini, Francesco Feliziani

**Affiliations:** 1 Institute for Veterinary Medical Research, Hungária krt. 21, 1143 Budapest, Hungary; tamas.vivien@vmri.hu (V.T.); meszaros.istvan@vmri.hu (I.M.); olasz.ferenc@vmri.hu (F.O.); zadori.zoltan@vmri.hu (Z.Z.); magyar.tibor@vmri.hu (T.M.); 2 Istituto Zooprofilattico Sperimentale Umbria-Marche “Togo Rosati”, Via Gaetano Salvemini, 1, 06126 Perugia, Italy; c.righi@izsum.it (C.R.); f.derrico@izsum.it (F.D.); c.casciari@izsum.it (C.C.); f.feliziani@izsum.it (F.F.)

**Keywords:** African swine fever, vaccine, CRISPR-Cas9, MGF 110-11L

## Abstract

African swine fever (ASF) is a highly lethal hemorrhagic viral disease that causes extensive economic and animal welfare losses in the Eurasian pig (*Sus scrofa*) population. To date, no effective and safe vaccines have been marketed against ASF. A starting point for vaccine development is using naturally occurring attenuated strains as a vaccine base. Here, we aimed to remove the multigene family (MGF) 110 gene of unknown function from the Lv17/WB/Rie1 genome to improve the usability of the virus as a live-attenuated vaccine, reducing unwanted side effects. The MGF 110-11L gene was deleted using the CRISPR/Cas9 method, and the safety and efficacy of the virus were tested in pigs after isolation. The vaccine candidates administered at high doses showed reduced pathogenicity compared to the parental strain and induced immunity in vaccinated animals, although several mild clinical signs were observed. Although Lv17/WB/Rie1/d110-11L cannot be used as a vaccine in its current form, it was encouraging that the undesirable side effects of Lv17/WB/Rie1 at high doses can be reduced by additional mutations without a significant reduction in its protective capacity.

## 1. Introduction

African swine fever (ASF) is a highly lethal hemorrhagic viral disease infecting the Eurasian pig (*Sus scrofa*), resulting in significant economic losses. ASF was first identified in Kenya in 1921 [1,2,3]. The disease is endemic in sub-Saharan countries but is spreading rapidly worldwide [4,5]. The ASF mortality rate is 100% in domestic pigs and wild boars; however, African *Suidae* species show subclinical symptoms and serve as viral reservoirs [6]. Despite decades of efforts, there is currently no effective vaccine against ASF [7]. ASF virus (ASFV) is a large, enveloped, double-stranded DNA virus. This virus is a member of the *Asfarviridae* family [8]. Its linear genome is 170–194 kbp, depending on the viral strain, and contains 170–190 open reading frames (ORFs). Approximately half of the encoded proteins have been experimentally identified; however, the functions of many of these proteins remain unknown [9,10,11]. The ASFV genome contains a relatively conserved central region. The genes in this genome section are primarily associated with replication. The central region is flanked by the left and right variable regions (LVR and RVR, respectively) with long repeated sequences. A comparison of different isolates revealed a high degree of variation in the number of genes in various regions [12,13]. In addition, most of the differences appear in the LVR (8–20 kb) [14]. Most of the genes in the variable regions belong to multigene families (MGFs).

MGFs can be classified into five families, namely MGF 100, MGF 110, MGF 300, MGF 360, MGF 505, and MGF 530 [13]. Members of MGF 300, 360, and 505 have a very similar N-terminal region [15] and many conserved sequence motifs among the MGF genes within a group [16]. Sequence analysis suggests that gene duplication drives the development of MGF genes [12]. One of the most characteristic changes in the ASFV genome during evolution is the appearance of gains or losses in the copy number of MGF proteins [16].

Although many of the functions of MGFs are still poorly understood, MGF proteins reportedly play essential roles in the viral life cycle and during in vitro and in vivo infections, including transcription, translation, virulence, and immune escape [17,18,19]. For example, it has been confirmed that MGF 360/530 genes could suppress type I interferon (IFN) response [17]. In another experiment, a mutant virus lacking three members of both (MGF 360 and MGF 530) gene families was shown to replicate in macrophage cultures but was avirulent in swine [18]. However, variants lacking the whole MGF 110 can replicate ex vivo at high titers and prove virulent; therefore, the members of the MGF 110 family do not seem to be essential for either in vitro replication or in vivo infection in domestic pigs [20].

The MGF 110 family consists of thirteen genes that are paralogs to each other. All the family members are transcribed exclusively on the reverse strand. Paralogs show a high degree of variation between strains with numerous insertions, deletions, and, in many cases, fusions [21]. Serial passaging of the virus in tissue culture often causes the loss of members of the MGF 110 family [12,22,23].

Even among naturally occurring attenuated strains, some strains lack the MGF 110 genes. For example, thirteen of the 26 genes missing from the viral genome of the Estonia 2014 strain belong to the MGF 110 (-1L-14L) multigene family [24]. The OURT 88/3 strain also lacks the MGF 110-4L-7L and -12-13L genes [13,25].

The properties of MGF 110 proteins have mainly been studied through experiments with deletion mutants, and our knowledge of their function is quite limited. *MGF 110-1L* is the only member of the MGF 110 family present in all ASFV isolates, yet it does not affect virulence [26]. MGF 110-4L and -6L are localized to the pre-Golgi compartments. These two proteins may be involved in ER rearrangements and impair the ER’s ability to synthesize proteins involved in cytokine production or antigen presentation [27]. The *MGF 110-5L-6L* gene is not involved in the development of clinical symptoms in swine [28]. MGF 110-7L activates the PERK/PKR-IF2a pathway, influencing host gene translation and inhibiting stress granule formation [29]. Deletion of the *MGF 110-9L* gene from a highly virulent strain results in partial attenuation of the virus, which is somewhat contradictory to earlier findings [30] that MGF 110 genes are not necessary for infectivity or virulence in pigs [20].

ASFV Lv17/WB/Rie1 was isolated in 2017 from hunted wild boar in Latvia. It replicates in peripheral blood mononuclear cells in the absence of hemadsorption (HAD). Lv17/WB/Rie1 can be characterized as a non-hemadsorbing (non-HAD), naturally attenuated isolate, and it contains a unique single nucleotide deletion inside the *EP402R* gene coding the CD2-like protein. This mutation results in a frameshift and early termination, likely creating a nonfunctional protein [31]. Domestic pigs infected with Lv17/WB/Rie1 either develop mild clinical signs (appearance of cyanosis in the ears and swelling joints), or the infection remains subclinical. In most cases, only short-term viremia can be detected, and the animals develop high anti-ASFV antibody titers. However, infected animals shed the virus in such amounts that in-contact pigs can be infected [32]. This strain has been tested as a potential oral vaccine for wild boars; immunization can confer 92% protection against virulent ASFV [33].

We hypothesized that deleting one of the MGF 110 genes in the Lv17/WB/Rie1 genome could improve the applicability of the virus as a live-attenuated vaccine by reducing unwanted side effects. *MGF 110-11L* was selected for deletion owing to its unique and uncharted characteristics. To test our hypothesis, the present study aimed to evaluate the safety and efficacy of the modified Lv17/WB/Rie1 virus as a vaccine candidate in pigs against experimental challenge by the virulent Armenia/07 ASFV. Our study contributes to a better understanding of the function of MGF 110 proteins and may help design safer and more effective ASFV vaccines.

## 2. Materials and Methods

### 2.1. Plasmid Design and Assembly

The Lv17/WB/Rie1/d110-11L construct was created by deleting the *MGF 110-11L* gene from the LV17/WB/Rie1 strain [31]. The *MGF 110-11L* gene was replaced with an enhanced green fluorescent protein (eGFP) under the control of the p72 promoter of ASFV.

To create the recombinant transfer plasmid (p14L-eGFP), the pUC19 vector (Thermo Fisher Scientific, Waltham, MA, USA) was used as the backbone. The recombination cassette contained a left homologous arm (1108 bp), a p72 promoter of ASFV, an eGFP gene, and a right homologous arm (1120 bp). p14L-eGFP was assembled from the linearized pUC19 transfer plasmid and three overlapping polymerase chain reaction (PCR) fragments (Table 1) of the recombination cassette using the Seamless PLUS Cloning and Assembly Kit (Thermo Fisher Scientific, Waltham, MA, USA).

Two gRNA plasmids were created by cloning one upstream and one downstream double-stranded protospacer oligonucleotide (Table 2) into the BbsI-digested pX330-DNLS1_2-NeoR plasmid [33].

### 2.2. Production and Isolation of Mutants

CRISPR/Cas9-mediated homologous recombination was used to generate the mutant virus, according to Borca et al. [34,35]. Macrophages were plated (5 × 10^6^/well) in six wells (Thermo Fisher Scientific, Waltham, MA, USA). Each well was infected with three MOI of Lv17/WB/Rie1 ASFV strains in 2 mL of RPMI medium (Thermo Fisher Scientific, Waltham, MA, USA). Next, 1.5 µg of transfer plasmid and 0.75–0.75 µL of gRNA plasmids were mixed in 150 µL of RPMI medium, and 10 µL of Fugene HD (Promega, Madison, WI, USA) transfection reagent was added to the mixed plasmids. After incubation for 10 min at room temperature, the mixture was added to the infected macrophages. The transfected porcine alveolar macrophages (PAMs) were incubated at 37 °C in 5% CO_2_ for 24 h_._

The fluorescent cells were manually isolated under a fluorescence microscope using a pipette. The isolated cells were frozen and thawed three times in 100 µL of RPMI medium; then, a 10-fold serial dilution was prepared, and 96-well plated (Thermo Fisher Scientific, Waltham, MA, USA) PAMs were infected with aliquots of the isolated viruses and incubated at 37 °C for 24 h. This procedure was repeated until the percentage of eGFP-expressing infected cells reached 100%. For the stock preparation, the PAMs were plated in 75 cm^2^ flasks (SARSTEDT AG&Co. KG, Nümbrecht, Germany) and infected with 100 µL of the isolated virus. The supernatant was collected and titrated after three days. The stock titer was calculated by fluorescent focus unit (FFU)-based determination.

### 2.3. Titer Determination

The 10-fold serial diluted LV17/WB/Rie1 or LV17/WB/Rie1/d110-11L cells were plated in 96-well PAM cultures. Twenty-four hours post-infection (hpi), the cells were fixed with 3% formaldehyde (VWR International, Radnor, PA, USA) and diluted in 1× PBS (Capricorn Scientific GmbH, Ebsdorfergrund, Germany). Immunofluorescent staining was performed with swine anti-ASFV polyclonal antibodies (Państwowy Instytut Weterynaryjny, Puławy, Poland) at 5000× dilution and with CF568-labeled anti-swine secondary antibodies (Biotium, Fremont, CA, USA) at 1000× dilution. The positive cells were detected using an Axio Observer D1 inverted fluorescence microscope (Carl Zeiss Ag. Oberkochen, Germany). The number of infected cells per well and the dilution rate allowed the calculation of the number of infectious viruses per milliliter and was expressed in FFU/mL.

### 2.4. Preparation and Culture of Porcine Alveolar Macrophages (PAMs)

The PAMs were prepared according to the OIE Manuals [36] and were stored in RPMI-1640 medium containing 30% fetal bovine serum (Sigma-Aldrich, Saint Louis, MO, USA) and 10% DMSO at −72 °C. The PAMs were cultured in a PAM culture medium that contained RPMI-1640 medium supplemented with 10% (*v*/*v*) fetal bovine serum, plus 1× antibiotic/antimycotic solution (Thermo Fisher Scientific, Waltham, MA, USA), and 2 mM of L-glutamine (Sigma-Aldrich, Saint Louis, MO, USA). The cells were incubated at 37 °C in 5% CO_2_.

### 2.5. Copy Number Determination

p72 gene-specific quantitative PCR (qPCR) was designed to quantify the ASFV genome. The qPCR (25 μL) was performed with 12.5 µL of DreamTaq PCR Master Mix (2×; Thermo Fisher Scientific, Waltham, MA, USA), 20× EvaGreenTM Dye (Biotium, Fremont, CA, USA), 1 µL of template DNA from the diluted supernatants, 1 µL of forward (F2: 5′-TACGTTGCGTCCGTGATAGG-3′), and 1 µL of reverse (R2: 5′-AGTTCGGATGTCACAACGCT-3′) primers at 1 µM concentration. The diluted supernatant of the infected PAMs was directly used as a template after heat treatment (72 °C, 20 min). The thermal reaction started with a 5 min pre-denaturation step at 95 °C, followed by 35 cycles consisting of denaturation at 95 °C for 30 s, annealing at 62 °C for 30 s, elongation at 72 °C for 35 s, and a post-elongation step at 72 °C for 5 min. The specificity of the qPCR was verified using melting curve analysis. The viral copy numbers were calculated using a standard curve with a 10-fold dilution of the purified amplicon as a template.

### 2.6. Sequencing

Whole genome sequencing was performed as described earlier [37]. The sequence was assembled using Geneious Prime 2019.2.3. For analysis, we used Bowtie2 and BBMap mapping methods with normal sensitivity. The LV17/WB/Rie1 strain (patented in Spain under reference PCT/2018/000069) was used as a reference.

### 2.7. Statistical Analysis

The Mann–Whitney U test was used to determine the significance level between the viral titers. The Kruskal–Wallis test was used to statistically analyze the specific infectivity. A *p*-value < 0.05 was considered statistically significant. The statistical analyses were performed using R software (version 4.2.2).

### 2.8. Animal Experiments

Thirteen 3-month-old castrated crossbred pigs (Danish Landrace × Danish Duroc), clinically healthy and devoid of ELISA ASFV antibodies (see Section 2.10), were housed in BSL3 animal facilities at the Istituto Zooprofilattico Sperimentale Umbria-Marche, Perugia, Italy. The pigs were fed twice daily with a diet for fattening and water ad libitum. The maintenance and experimental protocols were established following European legislation on the protection of animals used for scientific purposes, and the experiments were conducted with the approval of the Italian Ministry of Health (no. 424/2020-PR).

The assessment of the severity of injuries to establish the ‘human end point’ (HEP) was conducted using the welfare indicators described in the document approved by the competent national authorities for the implementation of Directive 2010/63/EU and taking due account of the guidelines in the ‘Working Document on a Severity Assessment Framework,’ drafted by an expert group. In particular, the HEP was carried out with the achievement of either a single item (hypothermia in 24 h; absence of food/water intake in 24 h; total immobility in 24 h) or in the case of an overall score > 17.

Before starting the experiments, the pigs were acclimated for seven days and then divided into three groups in separate stalls. The first two groups (Lv17d110-11L and Lv17) consisted of five pigs each, and the third group (control) consisted of three pigs. Group Lv17d110-11L was administered the vaccine candidate Lv17/WB/Rie1/d110-11L at a titer of 10^2^ FFU/2 mL, whereas group Lv17 was injected with a titer of 10^2^ FFU/2 mL of the Lv17/WB/Rie1 strain. The control group was unvaccinated. The inoculum was intramuscularly injected into the neck muscles of each pig (Table 3).

Twenty-one days post-vaccination (DPV), all the animals were challenged with the Armenia/07 strain of ASF. The virus was intramuscularly administered to the right side of the neck muscle. Each pig received 10 HAD_50_/2 mL via intramuscular injection.

Of note, a low dose of ASFV (10^1^–10^2^ HAD_50_) is reportedly sufficient to infect pigs and wild boars. Some of the animals infected did not show clinical signs indicative of ASFV and had almost no fever. On the contrary, the intramuscular injection showed clinical signs, including depression and fever, based on the injection doses [6].

The pigs were observed throughout the experimental period, and their clinical signs were recorded daily using a clinical score [6].

Serum and blood samples were collected at different time points post-vaccination (0, 7, 14, and 21 DPV) and after the infection challenge (0, 7, and 14 days post-challenge; DPC). Serum samples were used to detect antibodies against ASF, whereas blood samples were used for virological analyses using real-time PCR. At the end of the experiments, the pigs were euthanized, and necropsies were performed.

### 2.9. Collection of Blood Samples

Approximately 7 mL of blood was collected from the jugular vein of each pig. Disposable needles and vacutainer tubes were used for all the animals (Kima, Padova, Italy). The samples were then transferred to the laboratory and centrifuged at 850× *g* for 30 min at 4 °C to extract the serum for serological testing. Afterward, all the samples were stored at −20 °C for further serological studies.

### 2.10. ELISA Test

A commercial ELISA kit (Ingenasa, Ingezim PPA Compact, Madrid, Spain) was used to test the collected sera following the protocols supplied with the kits. The results were expressed according to the manufacturer’s instructions. The microplates were read using an automated plate reader (Infinite F50, Tecan AG, Männedorf, Switzerland), and the data were analyzed with the Magellan software (Tecan AG, Männedorf, Switzerland).

### 2.11. Real-Time PCR

Viral DNA was extracted from blood samples using a High Pure PCR Template Preparation Kit (Roche Diagnostics Deutschland, Mannheim, Germany) following the manufacturer’s instructions. To assess the presence of ASF, real-time PCR was performed as described in the Manual of Diagnostic Tests and Vaccines for Terrestrial Animals.

## 3. Results

### 3.1. Properties of MGF 110-11L Gene

**Figure 1:**
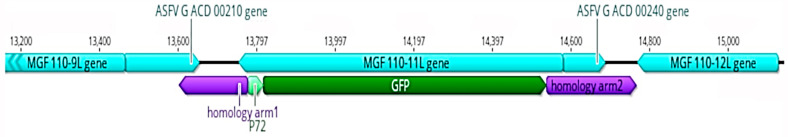
Schematic representation of the construction of MGF 110-11l deletion mutant. 760 bp of the 825 bp of the *MGF 110-11L* gene were replaced by the p72 promoter-controlled eGFP reporter gene.

**Figure 2:**
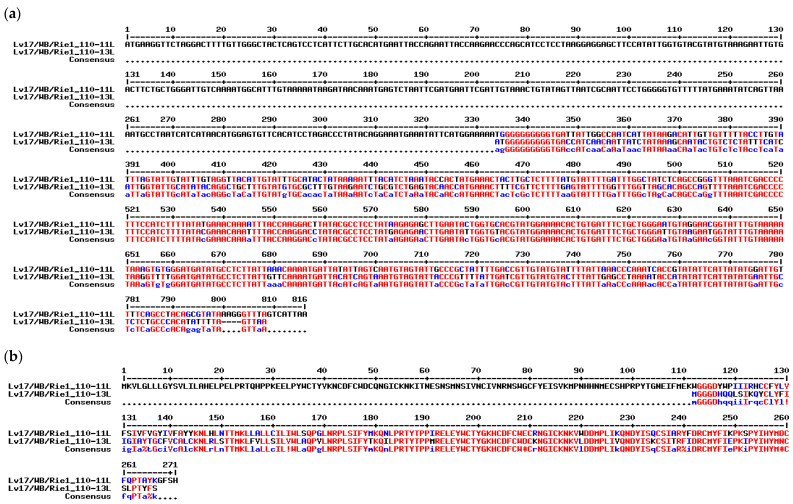
Nucleotide (**a**) and amino acid (**b**) differences between MGF 110-11L and MGF 110-13L of Lv17/WB/Rie1. Alignments were performed using the Multalin interface [38].

The *MGF 110-11L* gene has very specific properties in different strains; it can fuse with adjacent MGF 110 genes (e.g., MWI_LiL_20_1_1983: 9L/11L fusion protein; NHV: fusion between complete MGF 110-14L, -13L amino-terminus, and -11L carboxy-terminus) or have several large in-frame deletions [21]. It is one of the most divergent members of the MGF 110 family, and its closest relative within this group is the *MGF 110-13L* gene, with approximately 80% nucleotide and 69% amino acid sequence identity (Figure 1 and Figure 2a,b)

The *MGF 110-11L* has another interesting feature: it contains a homopolymeric G/C region with very high variability in its length. Comparing the MGF 110-11L sequences retrieved from GenBank revealed four 19 bases-long G/C stretches in the homopolymeric region (Table 4).

The role of this homopolymer region and the cause of the variance are unknown and puzzling because one nucleotide insertion/deletion in the G/C region leads to a frameshift in the ORF and causes significant variation in the length of the gene product.

### 3.2. Generation and Sequencing the Lv17/WB/Rie1/d110-11L Virus

The interesting features of MGF *110-11L* and the scarcity of biological data on this gene prompted us to investigate its function by deleting it from the Lv17/WB/Rie1 attenuated strain. The deletion of the *MGF 110-11L* was achieved using the CRISPR/CAS9-aided intercellular homologous recombination by substituting most of the gene (760 of the 825 bp ORF were deleted) with the p72 promoter-controlled eGFP gene (Figure 1). The transfer plasmid and CRISPR/Cas9 plasmids expressing both gRNAs and Cas9 proteins were co-transfected into Lv17/WB/Rie1-infected PAMs. The recombinant virus was isolated from PAMs expressing the eGFP reporter gene. After isolation and stock titration, we found that the mutant virus titer was in a similar range (3 × 10^6^ FFU/mL) to that of the parental virus (7.2 × 10^6^) grown under similar conditions. The Lv17/WB/Rie1/d110-11L viral stock was sequenced using the Illumina platform. Approximately 26 million viral reads were assembled, resulting in 42,000 average viral genome coverage. Fourteen unexpected point mutations and ambiguities were detected in different regions of the Lv17/WB/Rie1/d110-11L virus compared with the parental genome (Table 5). Two mutations affected the regulatory regions: one was a synonymous mutation, four caused amino acid substitutions, three caused early protein chain termination, and four ambiguities were detected, suggesting the presence of quasispecies in the viral population.

### 3.3. Replication of Lv17/WB/Rie1/d110-11L in Cultured PAMs

**Figure 3:**
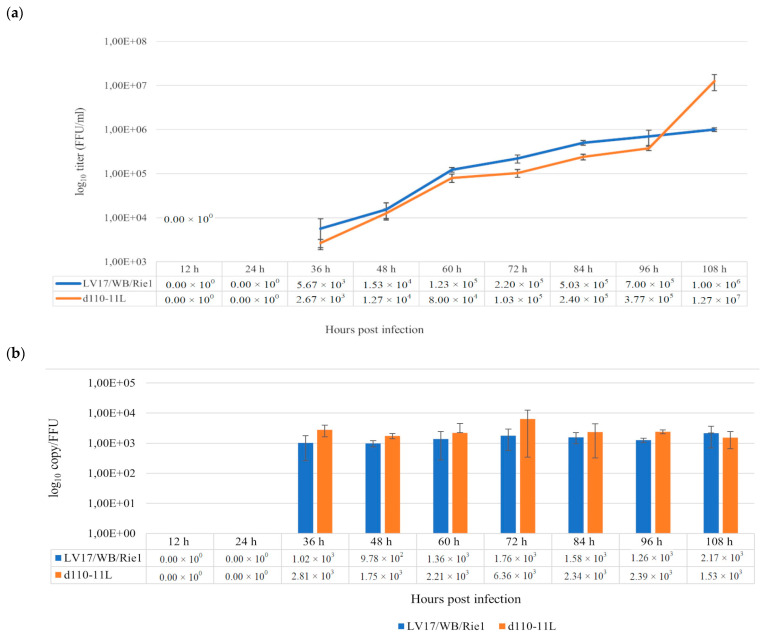
Growth characteristics of parental LV17/WB/Rie1 and the mutant LV17/WB/Rie1/d110-11L viruses. Porcine alveolar macrophages were infected (MOI = 0.001), samples were collected every 12 h from 12 to 108 h, and virus yields were titrated. (**a**) Means and standard errors of three parallel experiments. (**b**) Changes in specific infectivity (calculated as the ratio of genome copy/mL to FFU/mL).

To investigate the ex vivo replication features of the d110-11L mutant, PAMs were infected with d110-11L and LV17/WB/Rie1 as controls at an MOI of 0.001 and sampled at 0, 12, 24, 36, 48, 60, 72, 84, 96, and 108 hpi. The collected samples were then titrated in PAMs, and qPCRs were performed to determine the viral copy numbers in the aliquots. There were no significant differences in the growth kinetics of the mutant and parental viruses (36 h: *p* = 0.1642; 48 h: *p* = 1; 60 h: *p* = 0.07652; 72 h: *p* = 0.08086; 84 h: *p* = 0.08086; 96 h: *p* = 0.08086) up to 108 hpi, although the infective virus titer of d110-11L remained slightly but consistently below that of the parental strain. However, at 108 hpi, we observed a sudden jump in the infectious titer of d110-11L, with virus yields exceeding that of the LV17/WB/Rie1 virus by a factor of 12 (1.27 × 10^7^ FFU/mL versus 1 × 10^6^ FFU/mL; Figure 3a,b). Since the specific infectivity of the viruses did not change significantly during the last 24 h, this difference does not seem to be an artifact. It could be a consequence of slower growth and potentially higher yield of d110-11L.

The specific infectivity of both viruses slightly exceeded 10^3^ genome copies/FFU but remained relatively constant over the study period; the specific infectivity of both viruses did not differ significantly at different time points (Lv17/WB/Rie1: *p* = 0.458; Lv17/WB/Rie1/d110-11L: *p* = 0.5278). These results suggest that deletion of the MGF 110-11L gene and additional mutations did not significantly affect the specific infectivity of the LV17/WB/Rie1 virus in the PAMs.

### 3.4. In Vivo Characteristics of Lv17/WB/Rie1/d110-11L as a Vaccine Candidate

To compare whether the newly introduced mutations reduce the clinical signs of the parental virus at low doses (≥10^2^ FFU), two groups of animals were vaccinated with 10^2^ FFU Lv17/WB/Rie1 and Lv17/WB/Rie1/d110-11L viruses. During the vaccination period, the inoculated groups developed a mild clinical form of ASF. However, no severe clinical forms of ASF developed in the inoculated groups during the vaccination. However, two pigs in group Lv17d110-11L showed mild clinical symptoms such as diarrhea (pig #1; 13 DPV) and a caseous skin lesion on the right hind limb (pig #2; 11 DPV). Furthermore, two pigs in group Lv17 showed tremors (pigs #9, 10; 7–14 DPV), and one animal showed mild diarrhea (pig #10; 8 DPV).

**Figure 4:**
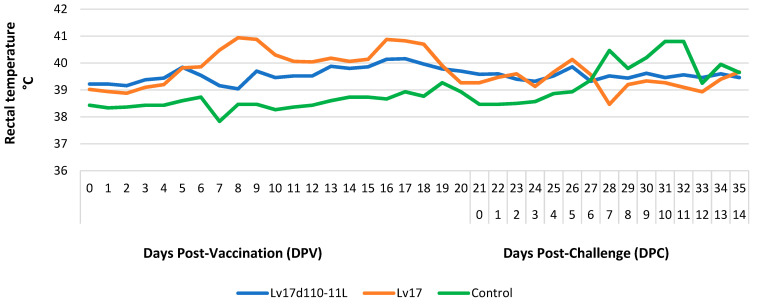
Rectal temperatures recorded in different groups injected with Lv17/WB/Rie1/d110-11L and Lv17/WB/Rie1 of African swine fever virus (ASFV) and challenge-infected with Armenia/07 of ASFV.

Following the challenge infection, the pigs in group Lv17d110-11L showed ecchymosis of the ear pinna (#1, #2; 0 DPC). Moreover, fever (>40.5 °C) was detected only in group Lv17 after 7 DPV. In the control group, fever was evident at 7 DPC (Figure 4).

**Figure 5:**
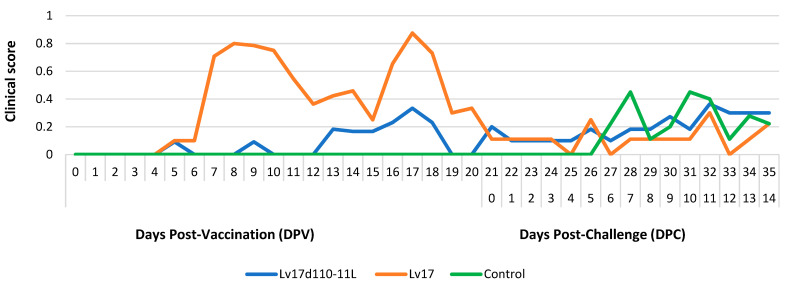
Clinical scores that were detected in different groups injected with Lv17/WB/Rie1/d110-11L and Lv17/WB/Rie1 of African swine fever virus (ASFV) and challenge-infected with Armenia/07 of ASFV.

During the entire vaccination period, the clinical score of group Lv17d110-11L remained below 0.5, in contrast to that of group Lv17, where the clinical score was under 1 at 18 DPV. Following the challenge infection, the clinical scores of all the animals in both groups were below 0.5 (Figure 5).

Three pigs suddenly died during the study. In particular, two pigs (#6 and #10) in group Lv17 died 19 days after vaccination, whereas one pig (#13) from the control group died 11 days after the challenge infection. The remaining pigs were euthanized at the end of the experiment.

Necropsy studies in group Lv17d110-11L revealed lesions characterized by mild lymph node hyperplasia, pleural adhesions, fibrinous pericarditis, hemorrhagic petechiae of the tricuspid valve, and endocarditis with thickening of the chordae tendineae. In group Lv17, lymph node lesions of enlargement and hemorrhagic, pleural adhesion, splenomegaly, superficial point hemorrhages of the kidney, diffuse hemorrhages of the skin, and hemorrhagic enteritis of the small intestine were observed. In addition, a circular necrotic lesion located in the elbow region was observed in one pig. In contrast, in the control group, lesions in the lymph nodes were represented by enlargement and hemorrhage, petechial hemorrhages of the palatine tonsil, splenomegaly, kidney superficial point hemorrhages, pericardial effusion, and pleural adhesions (Table 6).

The inoculated groups (Lv17d110-11L and Lv17) evidenced antibodies at 14 DPV. These positive results persisted until the end of the experiment. In contrast, the control group showed antibodies for the first time at 14 DPC (Table 7).

The first positive results were detected in Groups Lv17d110-11L and Lv17 by real-time PCR at 7 DPV. These positions persisted until 0 DPC. In group control, positive results were first detected by real-time PCR at 7 DPC, and the animals remained positive until the end of the experiment (Table 8).

## 4. Discussion

ASF remains one of the most concerning viral diseases affecting domestic pigs and wild boars, causing a significant economic burden. Following recent outbreaks in Asia and Europe, infections have emerged in America. Effective vaccines are urgently needed to prevent the spread of the virus and combat the pandemic.

The Lv17/WB/Rie1 strain is a seemingly suitable candidate for developing an effective live viral vaccine. Consequently, we genetically modified the Lv17/WB/Rie1 strain by deleting one of the MGF 110 genes and evaluated the suitability of the mutant as a vaccine.

Although naturally occurring, low-virulence strains offer an obvious basis for developing an effective live-attenuated vaccine, deletion of the genes involved in virulence and immune response can lead to unpredictable and unexpected results. For instance, the deletion of virulence-related genes (DP71L and DP96R) from the OUR T88/3 attenuated isolate reduced its ability to defend against the virulent virus, while the deletion of genes (A238L, A224L, EP153R, and A276R) in the low-virulence NH/P68 resulted in a complete failure to protect pigs or in the loss of cross-protection. To curtail the possibility of similar failures, we introduced mutation in an MGF 110 gene, which we expected to have potentially modest effects on LV17/WB/Rie1 and not overly attenuate the virus [6].

Although the exact function of MGF 110-11L is still unknown, some members of the MGF 110 family have immunosuppressive properties [27,29]. The interesting feature of MGF 110-11L is that it contains a long homopolymeric C/G region that has an important regulatory role in cellular organisms and other viruses. For example, G/C tracts affect regional gene expression in *C. elegans* [40]. Changing the length of the guanine tract of conserved sequence block 2 in human mitochondrial DNA resulted in transcription termination [41]. PolyC-binding proteins are involved in mRNA stabilization, translational activation, and translational silencing, and their binding to single- and double-stranded DNA mediates gene expression [42]. In picornaviruses, the polyC tract is required for replication in immune cells, and deletion of the polyC tract is associated with a loss of virulence and pathogenicity but does not affect replicative fitness in vitro [43,44].

Therefore, we hypothesized that the removal of the MGF 110-11L gene could cause significant attenuation through pleiotropic effects by eliminating the protein, on the one hand, and the potential regulatory function of the polyC/G tract on the other. Indeed, we experienced attenuation, however, not to the extent expected. It should be noted that the deleted part of MGF 110-11L also contains the transcription start site of ACD 00240 ORF (determined in ASFV Georgia 2007/1 by Cackett et al., in 2022 [45], after the design of our constructs and mutants), and, therefore, it most probably interferes with its transcription.

With few exceptions, gene knockouts in ASFV are not associated with the appearance of a large number of mutations in the genome [26,28,46,47,48], and the emergence of three additional mutations is rare [49]. Most mutations (11) have been reported following simultaneous knockout of the *MGF 110-9L* and *MGF 505-7R* genes [50]. It is tempting to speculate that the simultaneous loss of function of a gene from the MGF 110 and MGF 505 families would trigger a series of compensatory mutations; however, this seems to contradict the fact that there is only one overlapping gene (*CP2475L*) on the list of 11 and 14 mutated genes. Lv17/WB/Rie1 remained stable in our laboratories after several passages in PAMs, and Lv17/WB/Rie1/d110-11L was an outstanding exception regarding genetic stability among the numerous Lv17/WB/Rie1 deletion mutants generated (manuscript in preparation).

However, after isolation, the sequencing of the Lv17/WB/Rie1/d110-11L revealed an unexpectedly high number of mutations in the viral genome outside the deleted region. Many mutated genes can be linked to processes that can potentially affect the immune system and virulence of the virus. Although the exact function of MGF 505-5R is unknown, other MGF 505 genes suppress the type I interferon response and increase virulence in pigs. Deletion of MGF 505-1R, -2R, and -3R suppresses the viral replication in macrophages and attenuates ASFV [51], while MGF 505-7R inhibits cGAS-STING signaling and reduces IFN-β production [52]. However, the exact unique MGF 505-5R mutation was detected in virulent Polish isolates [53], which contradicts the significance of this mutation. EP364R is also involved in the reduction of the IFN response. It acts as a phosphodiesterase, impairs STING self-aggregation by cleaving the 2′,3′-cGAMP, and inhibits the cGAS-STING pathway that stimulates type I IFN [54]. At least three of the affected genes are involved in viral transcription. pG1340L is similar to the large subunit of the early transcription factor VACV A7 [11]. pNP1450L is an RNAP-like enzyme that is homologous to the largest subunit of eukaryotic RNA polymerase II, whereas *I243L* encodes a homolog of transcription elongation factor S-II (TFIIS) [10,55]. Interestingly, eukaryotic TFIIS is a component of RNA polymerase II pre-initiation complexes and is essential for the efficient formation of active pre-initiation complexes. The first two proteins are components of the viral nucleoid [9]. *CP2475L* encodes pp220, which is the main component of the core-shell structure. It acts as a protein scaffold and mediates interactions between the different virion layers [56,57]. The role of the MGF 100-2L protein is likely related to virulence [45].

In any case, the deletion of the *MGF 110-9L* gene and additional mutations did not cause a significant disadvantage in the in vitro replication of the virus and caused a mild reduction in the symptoms of the parental virus (although not to the desired extent). How the additional mutations affect the phenotype cannot be deciphered without further studies. The in vivo testing of the vaccine candidate showed a clear reduction in pathogenicity and lethality compared with the parental strain. Furthermore, the inoculated animals developed protection. In fact, the administration of the challenge strain had much fewer adverse effects than those observed in the control animals that suffered the full effects of the disease, even though an acute form was not evident since a low infectious dose was inoculated.

Nonetheless, the results obtained in the present study cannot be considered satisfactory. In our case, several clinical symptoms were observed, which, although mild, were attributable to the administration of the candidate vaccine. Unfortunately, similar symptoms have been observed in previous experiments using reduced-virulence ASFV strains; therefore, Lv17/WB/Rie1/d110-11L cannot be used as a vaccine in its present form. However, it is certainly encouraging that the undesirable side effects of Lv17/WB/Rie1, which occur at high doses, can be reduced by additional mutations without significantly reducing the protective capacity. It is clear that the removal of MGF 110-11L alone is not sufficient to provide the required safety improvement for vaccine licensing; however, there is a strong possibility that the desired result can be achieved by additional or other targeted genetic modifications.

## 5. Conclusions

Lv17/WB/Rie1 is an attenuated non-hemadsorbing ASFV strain that causes very weak or unapparent clinical symptoms in domestic pigs when inoculated intramuscularly with a lower dose (10 FFU/2 mL) [32]. This promising vaccine candidate induces 92% protection against virulent ASF in wild boars [33]. However, in its present form, it cannot be used as a vaccine because infection with a higher dose (e.g., ≥10^2^ FFU /2 mL intramuscularly) may induce unwanted clinical signs of ASF in domestic pigs. It seems reasonable that, by targeted genome modification, unwanted side effects can be eliminated or diminished while maintaining the beneficial properties of the virus strain for vaccination. One of the genes selected to study the effects of MGF removal was *MGF 110-11L*. We found that deletion of the *MGF 110-11L* gene and additional mutations did not cause a significant disadvantage in in vitro viral replication compared to the Lv17/WB/Rie1 strain but showed a clear reduction in pathogenicity and lethality. Unfortunately, mild side effects were still observed; therefore, Lv17/WB/Rie1/d110-11L in its current form cannot be used as a vaccine. Our results suggest that further targeted genetic modifications can achieve the desired results.

## Figures and Tables

**Table 1:** Primers used for the amplicons to construct the transfer plasmid.

Covered and Amplified Region	Name of the Primer	Sequence
pUC19 + homology arm 1	reg1_d11_F	5′-ACGGCCAGTGAATTCGAGCTCGGTA CGTTATGTTGATAGTGTA-3′
homology arm 1 + p72 promoter	reg1_ d11_R	5′-ATATAATGTTATAAAAATAATTTATTGTT TTTATTAAATACGTATAAAGGGTTTAGTCATTAATAG-3′
p72 promoter + eGFP	reg2_d11_F	5′-TATTTAATAAAAACAATAAATTATT TTTATAACATTATATATGGTGAGCAAGGGCGAGGAGC-3′
homology arm 2 + eGFP	reg2_d11_R	5′-GCTACTCAGTCCTC ATTTTACTTGTACAGCTCGTCCATG-3′
eGFP + homology arm 2	reg3_d11_F	5′-GCATGGACGAGCTG TACAAGTAAAATGAGGACTGAGTAGCC-3′
pUC19 + homology arm 2	reg3_d11_R	5′-GGAAACAGCTATGACCATGAT TACGCCAAGCTTGCATGCCTCTATAAAGCAATACTGTC-3′

**Table 2:** Oligonucleotides used for gRNA cloning.

Name of the Oligonucleotide	Sequence
CRISPR11_p72F	5′-CACCG CTAAACCCTTTATACGCTGT-3′
CRISPR11L_p72R	5′-AAAC ACAGCGTATAAAGGGTTTAG C-3′
CRISPR11L_endF	5′-CACCG GTAATTCATGTGCAAGAATG-3′
CRISPR11L_endR	5′-AAAC CATTCTTGCACATGAATTAC C-3′

**Table 3:** African swine fever virus (ASFV) vaccine candidate and ASFV strain used in the experiment.

Group	No. of Pigs	Vaccine Candidate/ Strain	Virus Concentration in FFU * in One Dose (2 mL)	Inoculation Route
Lv17d110-11L	5	Lv17/WB/Rie1/d110-11L	10^2^	Intramuscular
Lv17	5	Lv17/WB/Rie1	10^2^	Intramuscular
control	3	Unvaccinated controls		

* FFU, fluorescent focus unit.

**Table 4:** G number variance in the G/C stretch of the *MGF 110-11L* gene of different African swine fever virus (ASFV) isolates. All sequences were collected from GenBank using nucleotide BLAST [39].

MGF 110-11L Gene	
Length of the Homopolymer G Region	Number of Isolates
4 bp	1
7 bp	1
8 bp	50
9 bp	23
10 bp	23
11 bp	17
12 bp	10
13 bp	21
14 bp	15
17 bp	1
19 bp	1
	Total: 163

**Table 5:** Comparison of the genomes of LV17/WB/Rie1/d110-11L and LV17/WB/Rie1 viruses.

Position in LV17/WB/Rie1 Genome	Change in d110-11L (Nucleotide)	Change in d110-11L (Amino Acid)	Gene	Other Information	Major Variants
11,363	C > T	-	*NCR*	promoter/regulator region of 285L gene	95.9%
38,598	G > A	Val > Ile	*MGF 505-5R*	both the original and the substituted aa has an aliphatic side-chain	95.6%
76,238	insertion of A	frameshift	*EP364R*	early termination, the length has reduced from 364 to 232 aa	85.4%
112,655	C > A	Lys > Asn	*G1340L*	basic to polar aa	70.3%
115,161	G > T	Gly > Cys	*G1211R*	apolar aa to polar aa	92.4%
119,295	C > Y	Gly > Gly/Asp	*CP2475L*	ambiguous aa	C/T = 56.8/41.2%
130,645	C > Y	Ala > Ala/Thr	*NP1450L*	ambiguous aa	C/T = 55.4/42.4%
134,614	G > R	Ala >Ala/Val	*NP419L*	ambiguous aa	A/G = 55.6/42.9%
166,363	G > K	Pro > Pro/Gln	*E146L*	ambiguous aa	G/T = 41.1/53.1%
171,591	insertion of A		*NCR*	promoter/regulator region of the I267L gene	73.7%
172,645	deletion of T	frameshift	*I243L*	early termination, length has reduced from 243 to 158 aa	70.0%
176,321	C > T	Asp > Asn	*I196L*	acidic to polar aa	98.4%
180,817	insertion of T	frameshift	*MGF 100-2L*	early termination, the length has reduced from 141 to 80 aa	69.7%
185,263	C > T	Glu > Glu	*DP71L*	synonymous mutation	85.6%

**Table 6:** Description of the macroscopic lesions observed in different groups injected with Lv17/WB/Rie1/d110-11L and Lv17/WB/Rie1 of African swine fever virus (ASFV) and challenge-infected with Armenia/07 of ASFV.

Group	Pig #	Lymph Nodes ^†^	Tonsils	Lungs	Spleen	Kidney	Heart	Skin ^††^	Intestine
Lv17d110-11L	1	-	-	-	-	-	-	-	-
	2	-	-	+/d	-	-	+/l	-	-
	3	+/m	-	-	-	-	+/l, n	-	-
	4	+/m	-	-	-	-	-	-	-
	5	-	-	-	-	-	+/o	-	-
Lv17	6	+/a	-	-	+/b	-	-	+/h	-
	7	+/a	-	-	+/b	-	-	-	-
	8	+/a	-	-	+/b	-	-	-	+/i
	9	+/a	-	-	-	+/e	-	-	-
	10	+/a	-	+/d	+/b	-	-	+/g	-
control	11	+/a	-	-	+/b	+/e	+/e	-	-
	12	+/a	+/f	-	+/b	+/e	+/e	-	-
	13	+/a	-	+/d	+/b	+/e	+/c	-	-

^†^ bronchial, submandibular, epigastric, perirenal, mesenteric, superficial inguinal; ^††^ head, limbs, buttocks, ears; a, enlargement and hemorrhagic; b, splenomegaly; c, pericardial effusion; d, pleural adhesions; e, superficial point hemorrhages; f, petechial hemorrhages; g, diffuse hemorrhages associated with a circular necrotic lesion the size of a coin located in the region of the elbow; h, diffuse hemorrhages; i, hemorrhagic enteritis of the small intestine; l, fibrinous pericarditis; m, mild hyperplasia; n, hemorrhagic petechiae of the tricuspid valve; o, endocarditis with thickening of the chordae tendineae.

**Table 7:** Antibody response of pigs immunized with different African swine fever virus (ASFV) strains and challenged with Armenia/07 of ASFV.

Strain/Controls	Days Post-Vaccination (DPV)			Days Post-Challenge (DPC)		
	0	7	14	0 *	7 **	14 ***
Lv17/WB/Rie1/d110-11L	-	-	5 (70.7%) ^a,b^	5 (72.2%)	5 (90.8%)	5 (95.6%)
Lv17/WB/Rie1	-	-	5 (78.2%)	3 (78.4%) ^c^	3 (89.8%) ^c^	3 (93.3%) ^c^
Controls	-	-	-	-	-	1 (80.37%) ^d^

* 21 DPV; ** 28 DPV; *** 35 DPV; ^a^ positive detection ratio obtained by commercial ELISA test (Ingenasa, Ingezim PPA Compact, Spain): ^b^ Number of pigs (average blocking percentage value); ^c^ two pigs died 19 days after the vaccination; ^d^ one pig died 11 days after challenge infection.

**Table 8:** Results of ASFV detection in pigs immunized with different African swine fever virus (ASFV) strains and challenged with Armenia/07 of ASFV by real-time PCR °.

Strain	Days Post-Vaccination (DPV)			Days Post-Challenge (DPC)		
	0	7	14	0 *	7 **	14 ***
Lv17/WB/Rie1/d110-11L	-	5 (35.2) ^a,b^	4 (37.4)	1 (37.41)	-	-
Lv17/WB/Rie1	-	5 (32.7)	3 (33.8)	1 (36.5) ^c^	-	-
Unvaccinated controls	-	-	-	-	2 (24.2)	2 (29.38) ^d^

° OIE Manual of Diagnostic Tests and Vaccines for Terrestrial Animals; * 21 DPV; ** 28 DPV; *** 35 DPV; ^a^ positive detection ratio obtained by real-time PCR; ^b^ number of pigs (average of threshold cycles value); ^c^ two pigs died 19 days after the vaccination; ^d^ one pig died 11 days after challenge infection.

## Data Availability

Not Applicable.
